# Remote proctoring for high-risk coronary interventions with mechanical circulatory support during COVID-19 pandemic and beyond

**DOI:** 10.1007/s00392-021-01890-3

**Published:** 2021-06-22

**Authors:** Felix J. Woitek, Stephan Haussig, Johannes Mierke, Axel Linke, Norman Mangner

**Affiliations:** Herzzentrum Dresden, Department of Internal Medicine and Cardiology, Technische Universität Dresden, Fetscherstr. 76, 01307 Dresden, Germany

**Keywords:** Mechanical circulatory support, Impella, Remote proctoring, High-risk PCI

## Abstract

Remote proctoring by advanced digital technologies may help to overcome pandemic, geographic, and resource-related constraints for mentoring and educating interventional cardiology skills. We present a case series of patients undergoing high-risk percutaneous coronary intervention (HR-PCI) with mechanical circulatory support (MCS) guided by remote proctoring to gain insights into a streaming technology platform with regard to video/audio quality, visibility of all structural and imaging details, and delay in transmission. According to our experience, remote proctoring appears to be a reliable, quick, and resource-conserving way to disseminate, educate and improve MCS-supported HR-PCI with implications far beyond the COVID-19 pandemic.

Sirs:

With the appearance of a novel coronavirus, severe acute respiratory syndrome coronavirus-2, and the consequential pandemic [1], strict private- and business-related travel restrictions became necessary and have been implemented. Interventional and surgical specialties particularly depend on practical education to provide high-quality care for patients. Mastering certain procedures is often realized by on-site proctoring by an experienced external operator; however, the travel ban has restricted those activities. On the other hand, many hospitals have reduced their elective program including cardiovascular procedures to provide structural and personal resources to handle patients with coronavirus disease 19 (Covid-19) [2]. Postponing procedures in the cardiovascular medicine has to balance the risks and benefits of this decision since some cardiovascular interventions in certain clinical situations are not truly elective. The European Association of Percutaneous Cardiovascular Interventions has provided a consensus document how patients and cardiovascular procedures should be prioritized [3]. According to these criteria, patients with acute coronary syndrome or coronary artery diseases having symptoms according to Canadian Cardiovascular Society class IV and/or require left main stem percutaneous intervention (PCI) or last-remaining vessel PCI should not be postponed and must be treated urgently. Those patients are often characterized by relevant comorbidities, reduced left ventricular ejection fraction and complex coronary artery disease fulfilling the criteria for high-risk (HR) PCI, thereby qualifying for short-term mechanical circulatory support (MCS) [4]. The Impella^®^ 2.5 and CP heart pumps are nowadays the most often used MCS in the setting of HR-PCI [5]. However, the application can be associated with substantial complications including bleeding, access site complications and stroke negatively affecting the outcome of those patients [5]. It has been shown that the establishment of a MCS program is characterized by a certain learning curve on both the operator and center level [4, 6].

Against this background, we used a remote proctoring system: (1) to test the feasibility of this system for remote proctoring of MCS-supported HR-PCI with regard to video/audio quality, visibility of all structural and imaging details, and delay in transmission and (2) to perform educational sessions on MCS-supported HR-PCI for physicians and technical staff.

The remote proctoring system was provided by TEGUS Medical (TEGUS Medical, Hamburg, Germany). It consists of the following hardware that is placed in the cath lab: (1) a 360° rotatable and 180° tiltable high definition PTZ network camera (1920 × 1080 resolution; with optical zoom and optimized framerate) which is mounted to a purpose built stand-alone freely moveable tripod, (2) a small form factor server, which enables data transformation and online access, and (3) a lightweight Bluetooth headset for audio communication with the operator (Fig. 1A). The proctor uses any conventional desktop computer to connect to the cath lab via an online platform developed for on-demand visual and acoustic live streaming (Fig. 1B). There is no recording, only livestreams are used with no sensible data storage. The online platform is programmed, provided and maintained by TEGUS Medical (Hamburg, Germany). Access to the platform is password secured and data transfer is encrypted. After logging into the platform, the proctor is able to navigate the camera inside the cath lab and zoom into any spot, e.g., the hands of the operator, the imaging screens or the Impella^®^ controller simply via a mouse click-to-move approach, e.g., clicking on the area of interest directly on the screen. The focus and brightness are controlled automatically to facilitate ease of use for the proctor; however, additional function buttons to pause audio transmission and manually adjust the focus and brightness are also provided. A “preset” function is also provided to enable quick movement between predefined views (Fig. 2).

**Fig. 1:**
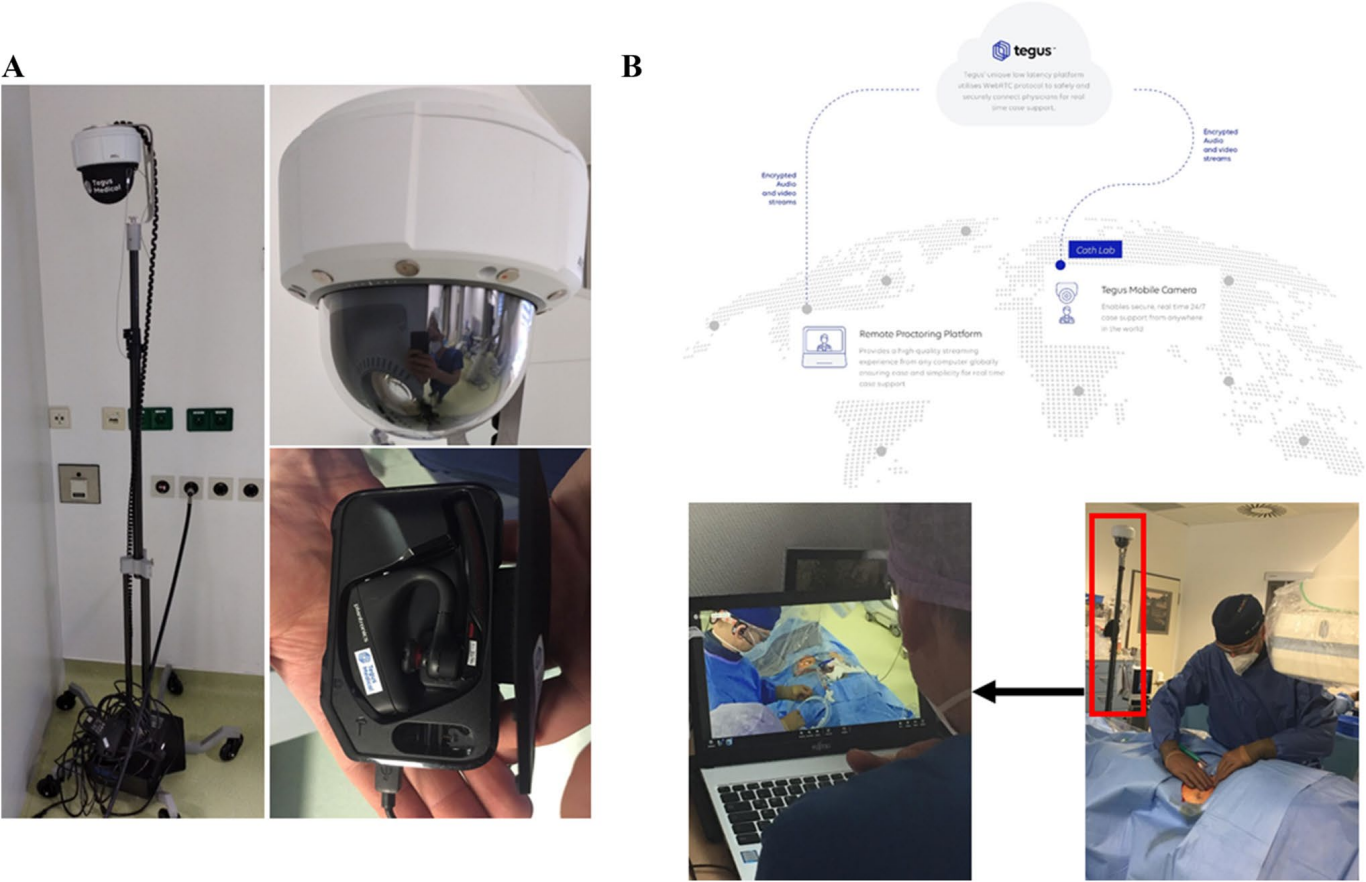
TEGUS remote proctoring system showing the stand-alone freely moveable tripod with focus on the high definition camera and the Bluetooth headset (**A**). The stand-alone freely moveable tripod is best positioned at the foot of the floating cath lab table (red box in the lower part of the **B**) providing the audiovisual connection to the proctor in the password secured and encrypted data transfer TEGUS platform (upper part of **B**)

**Fig. 2:**
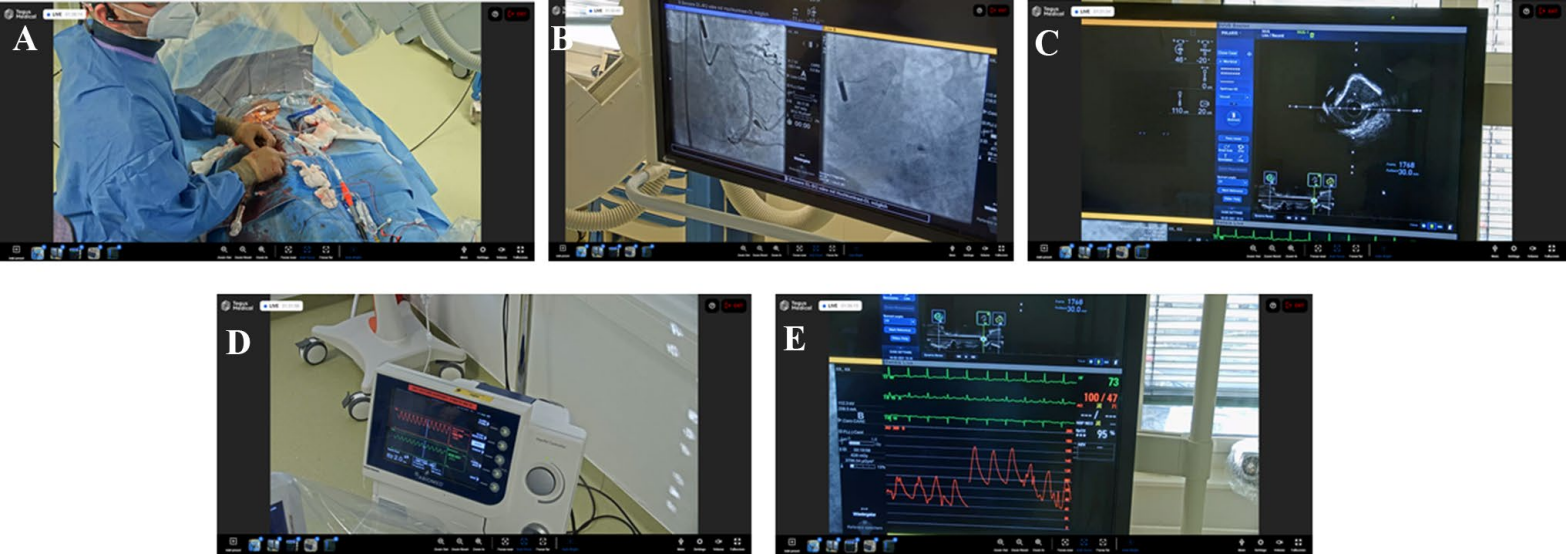
Screenshots from the TEGUS online platform of the operating field (**A**), angiography (**B**), intravascular ultrasound (**C**), the Impella^®^ controller (**D**), and hemodynamics (**E**)

We performed six HR-PCI cases with Impella^®^ support under remote proctoring. Patients are included the Dresden Impella Registry that has been approved by the Ethics Committee at TU Dresden (EK 457-122-014). Patient- and procedural details as well as in-hospital outcomes are outlined in the Table 1. Patients represented a typical cohort for HR-PCI with Impella 2.5 and a single access strategy used in all cases. Extensive lesion preparation including rotablation and cutting balloon PCI was performed. All procedures were successful without in-hospital complications and discharge to home the following day in the majority of patients.

**Table 1:** Baseline characteristics, procedural details and in-hospital outcomes

	Patient # 1	Patient # 2	Patient # 3	Patient # 4	Patient # 5	Patient # 6
Patient characteristics						
Age, years	86	89	86	67	81	86
Sex	male	male	male	male	male	male
Body mass index, (kg/m2)	26.8	22.9	26.1	32.5	34.4	32.7
Clinical presentation	Unstable angina pectoris	NSTEMI	Cardiac decompensation	Cardiac decompensation	NSTEMI	NSTEMI
Comorbidities	Arterial hypertension, status post 2-chamber-pacemaker due to sick sinus syndrome, previous stroke	Arterial hypertension, PAD, carotid disease, chronic renal disease (GFR 39 ml/min)	Arterial hypertension, PAD, Diabetes mellitus, COPD, chronic renal failure (GFR 61 ml/min)	Arterial hypertension, carotid disease, complex PAD with recent femoral-popliteal bypass left and lower leg amputation right, hip replacement, consequently poor mobility	Arterial hypertension, diabetes mellitus	Arterial hypertension, chronic renal failure (GFR 60 ml/min)
Previous myocardial infarction	No	No	No	No	No	No
Previous PCI	No	Yes (RCA 1 week before HR-PCI)	No	No	No	No
Previous cardiac surgery	No	No	No	No	No	No
LV-EF, %	40	25	17	23	65	35
Valvular heart disease	No	AS II°, MR II°	No	AS II°, TR II°	AS I°, MS II°, MR II°	No
Procedural characteristics						
Impella^®^	2.5	2.5	2.5	2.5	2.5	2.5
Access site	Right common femoral artery	Left common femoral artery	Right common femoral artery	Right common femoral artery	Right common femoral artery	Left common femoral artery
Single access strategy	Yes	Yes	Yes	Yes	Yes	Yes
Coronary artery disease	2-vessel disease	3-vessel disease	3-vessel disease	3-vessel disease	3-vessel diseases	3-vessel disease
Dominance	left	right	left	right	left	right
Left main > 50%	Yes (Medina 1-1-0)	Yes (Medina 1-1-1)	Yes (Medina 1-0-0)	Yes (Medina 1-1-1)	Yes (Medina 1-1-1)	Yes (Medina 1-1-1)
Proximal LAD > 75%	Yes	Yes	No	Yes	Yes	Yes
CTO	No	No	No	Yes (RCA)	Yes (RCA)	No
Target vessel(s)	Left main, LAD	Left main, LAD, Cx	Left main	Left main, LAD, Cx	Left main, LAD, Cx	Left main, LAD, Cx
Rotablation	No	No	No	Yes (left main/Cx)	Yes (left main/LAD + left main/Cx)	Yes (left main/LAD)
Cutting balloon	Yes	No	Yes	No	Yes	No
Bifurcation technique	Provisional Stenting left main	Mini-Crush left main	No	DK-Crush left main	DK-Crush left main	DK-Crush left main
Intravascular imaging use	No	No	No	No	IVUS	IVUS
Access site closure	MANTA 14F	MANTA 14F	MANTA 14F	MANTA 14F	MANTA 14F	MANTA 14F
Procedure duration, min	90	108	65	126	157	114
Contrast dye, cc	150	200	170	100	170	240
Fluoroscopy, min	14.2	11.2	9.1	29	40.3	25.4
In-hospital outcomes						
Death	No	No	No	No	No	No
Acute renal failure	No	No	No	No	No	No
Stroke	No	No	No	No	No	No
Major vascular complication	No	No	No	No	No	No
Major bleeding	No	No	No	No	No	No
Length of hospital stay after PCI, days	1	1	1	7	1	3

*NSTEMI* non-ST segment elevation myocardial infarction, *PAD* peripheral artery disease, *COPD* chronic obstructive pulmonary disease, *RCA* right coronary artery, *(HR)-PCI* (high-risk) percutaneous coronary intervention, *AS* aortic stenosis, *MR* mitral regurgitation, *TR* tricuspid regurgitation, *MS* mitral stenosis, *LAD* left anterior descending coronary artery, *Cx* circumflex coronary artery, *CTO* chronic total occlusion, *IVUS* intravascular ultrasound

The TEGUS remote proctoring system provided a stable and high-quality video and audio signal throughout all procedures from initial puncture till access site closure. With maximum zoom, the resolution was high enough to clearly identify the interventional equipment and angiography on the screen, in particular IVUS pictures were clearly visible. Not only the procedure, but also the preparation of the Impella^®^ heart pump and the controller could be supervised and in case of any alarms, advise for troubleshooting was given. In our setting, audio connection was only established between the proctor and the operator via a Bluetooth headset and not to the whole cath lab team. Audio connection is also possible via a remote loudspeaker which might have the advantage to provide direct advice to the unsterile cath lab staff, e.g., for adjustments at the Impella® controller. In one case, the hospital internet was disturbed; however, the integrated 4G mobile router maintained a stable connection between the proctor and operator. With both connections, no relevant lag in transmission was observed which is an important finding since in HR-PCI cases certain decisions have to be made immediately. Moreover, physicians and staff members attending as invited viewers on the secured website also reported on high-quality audio and video signals without lag in transmission indicating that the TEGUS system is not only a viable option for remote proctoring but also for streaming of educational sessions performed by an operator and potentially commented by the proctor.

Remote proctoring has been developed in operative disciplines [7], and has recently been described in a structural intervention case performing reverse LAMPOON (intentional laceration of the anterior mitral valve leaflet to prevent left ventricular outflow obstruction)-assisted transcatheter mitral valve implantation [8]. The TEGUS system was specifically developed for endovascular interventions and has been primarily introduced in a neurovascular scenario [9, 10]. To our knowledge, this is the first report on a series of remote proctored MCS-supported HR-PCI cases with the TEGUS system suggesting the applicability of this approach. Interventional cardiology and cardiac catheterization expertise is critical to the success of a percutaneous MCS program. As mentioned before, a significant learning curve exists and investment in training of the operator and the whole team is necessary to improve patient care and hemodynamic support by the MCS [4].

Our report has certain limitations: (1) Interventions and proctoring were performed by two experienced interventional cardiologists working together for several years. Therefore, proctoring between two unknown persons might be different. (2) Cases have been discussed face-to-face between the operator and proctor before. In real remote proctoring cases, patient’s characteristics, diagnostic findings, and the procedural strategy should be discussed in advance via a virtual meeting. (3) Stable internet connections are a prerequisite for this kind of proctoring with technical network requirements provided by TEGUS Medical.

Remote proctoring appears to be a reliable, quick, and resource-conserving way to disseminate, educate and improve MCS-supported HR-PCI in particular and interventional cardiology skills in general. The application of this approach is far beyond the COVID-19 pandemic.

## Abbreviations

HR-PCI: High-risk percutaneous coronary intervention
MCS: Mechanical circulatory support
